# Rod photoreceptor-specific deletion of cytosolic aspartate aminotransferase, GOT1, causes retinal degeneration

**DOI:** 10.3389/fopht.2023.1306019

**Published:** 2023-12-05

**Authors:** Shubha Subramanya, Moloy T. Goswami, Nicholas Miller, Eric Weh, Sraboni Chaudhury, Li Zhang, Anthony Andren, Heather Hager, Katherine M. Weh, Costas A. Lyssiotis, Cagri G. Besirli, Thomas J. Wubben

**Affiliations:** 1 Department of Ophthalmology and Visual Sciences, University of Michigan, Ann Arbor, MI, United States,; 2 Department of Molecular & Integrative Physiology, University of Michigan, Ann Arbor, MI, United States,; 3 Department of Internal Medicine, Division of Gastroenterology and Hepatology, University of Michigan, Ann Arbor, MI, United States,; 4 Rogel Cancer Center, University of Michigan, Ann Arbor, MI, United States

**Keywords:** photoreceptor, GOT1, malate-aspartate shuttle, redox, TCA cycle, metabolism

## Abstract

Photoreceptor cell death is the cause of vision loss in many forms of retinal disease. Metabolic dysfunction within the outer retina has been shown to be an underlying factor contributing to photoreceptor loss. Therefore, a comprehensive understanding of the metabolic pathways essential to photoreceptor health and function is key to identifying novel neuroprotective strategies. Glutamic-oxaloacetic transaminase 1 (*Got1*) encodes for a cytosolic aspartate aminotransferase that reversibly catalyzes the transfer of an amino group between glutamate and aspartate and is an important aspect of the malate-aspartate shuttle (MAS), which transfers reducing equivalents from the cytosol to the mitochondrial matrix. Previous work has demonstrated that the activity of this enzyme is highest in photoreceptor inner segments. Furthermore, *ex vivo* studies have demonstrated that the retina relies on aspartate aminotransferase for amino acid metabolism. Importantly, aspartate aminotransferase has been suggested to be an early biomarker of retinal degeneration in retinitis pigmentosa and a possible target for neuroprotection. In the present study, we characterized the effect of *Got1* deletion on photoreceptor metabolism, function, and survival *in vivo* by using a rod photoreceptor-specific, *Got1* knockout mouse model. Loss of the GOT1 enzyme from rod photoreceptors resulted in age-related photoreceptor degeneration with an accumulation of retinal aspartate and NADH and alterations in the expression of genes involved in the MAS, the tricarboxylic acid (TCA) cycle, and redox balance. Hence, GOT1 is critical to *in vivo* photoreceptor metabolism, function, and survival.

## 1 Introduction

Photoreceptor (PR) cell death is the ultimate cause of vision loss in many retinal degenerative diseases. Despite recent advances in gene therapy for inherited retinal diseases and complement therapies for late-stage, nonexudative age-related macular degeneration, an unmet need to develop PR neuroprotective therapies to prevent vision loss still exists due to the limitations in these treatment strategies (1, 2). The retina is one of the most metabolically active tissues in the body due largely to the maintenance of the dark current as well as the constant renewal of outer segments of PR cells (3, 4). Metabolic dysregulation and oxidative stress have been identified as unifying mechanisms in PR death (5, 6), and mutations in ubiquitously expressed metabolic enzymes have been associated with isolated retinal degenerations, suggesting that metabolic homeostasis is essential for long-term PR survival. To this end, understanding retinal metabolism and how it is altered in disease has been identified as a priority area of research as unraveling the metabolic and redox pathways integral to PR health may identify novel targets for neuroprotective strategies (7).

The immense metabolic demands of PR cells require the shuttling of reducing equivalents (e.g. NADH) around the cell to maintain metabolic flux and homeostasis (8). The malate-aspartate shuttle (MAS) transfers reducing equivalents from the cytosol to the mitochondria to support oxidative phosphorylation (9), regenerates NAD^+^ to support glycolysis (8), and can support redox balance via the production of nicotinamide adenine dinucleotide phosphate (NADPH) through malic enzyme 1 (ME1) (9). PRs express high levels of MAS components (10), and their activities are highest in PR inner segments (11). Previous work has demonstrated that the MAS is essential for both retinal function and glucose oxidative metabolism, and also serves to protect glutamate from oxidation in the retina (8, 12). Glutamic-oxaloacetic transaminase 1 (*Got1*) encodes for cytosolic aspartate aminotransferase, an essential enzyme of the MAS, which catalyzes the reversible transfer of an amino group between glutamate and aspartate. *Ex vivo* studies have demonstrated that the retina relies on aspartate aminotransferases for amino acid metabolism (13, 14), and GOT1 has been shown to be a biomarker of early retinal degeneration in retinitis pigmentosa (15), suggesting a possible target for therapeutic approaches.

To develop an *in vivo* understanding of the importance of this pathway in PRs, we generated a mouse model lacking *Got1* specifically in rod PRs. We used this novel transgenic mouse model coupled with metabolomics methodologies to demonstrate that GOT1 activity is critical to PR metabolism, function, and survival. Loss of the GOT1 enzyme from rod PRs resulted in age-related PR degeneration with significant effects on both retinal aspartate and NADH metabolism as well as the expression of genes involved in mitochondrial function and redox balance.

## 2 Materials and methods

### 2.1 Animals

Mice were treated in accordance with the Association for Research in Vision and Ophthalmology Statement for the Use of Animals in Ophthalmic and Vision Research and with approval from the Institutional Animal Care & Use Committee at the University of Michigan (Protocol number: PRO00011133). All mice were housed at room temperature in 12-hour light/12-hour dark cycles with free access to food and water. Male and female mice were randomly allotted to experimental groups. Animals were maintained on a C57BL/6 background and were confirmed to not carry the *rd8* mutation. Mice harboring loxP sites flanking exon 3 of the *Got1* gene were a generous gift from Dr. Costas Lyssiotis and originally created by Ozgene (Perth, WA, Australia). Specifically, the *Got1* conditional knock-out allele was generated by flanking exon 3 with loxP sites via gene targeting in mouse embryonic stem cells (16). Gene targeted embryonic stem cell clones were identified and then injected into goGermline blastocysts (17). Male goGermline mice were bred to C57BL/6 females to establish heterozygous germline offspring on C57BL/6 background. Cre-mediated deletion of exon 3 of *Got1* is expected to result in the generation of a transcript encoding for a protein product with a predicted frameshift mutation (p.Val101Argfs*34). Any transcripts which escape nonsense mediated decay are predicted to result in translation of a partially complete GOT1 protein lacking most of the amino acid residues required for enzymatic function (18). These mice were crossed to mice harboring a Cre recombinase under the control of the rhodopsin promoter (19) to create animals with conditional deletion of *Got1* from rod PRs, specifically (*Got1*^*fl/fl*^*; Rho-Cre*^+^, cKO). Animals expressing Cre recombinase under the same promoter, but lacking the floxed *Got1* allele, were used as control animals (*Got1*^*wt/wt*^*;Rho-Cre*^+^, WT).

### 2.2 Immunofluorescence

Mouse eyes were enucleated and fixed in 10% neutral buffered formalin (Epredia, Netherlands B.V.; Cat# 511201) overnight, embedded in paraffin, and sectioned to 4 μm thickness. Sections were de-paraffinized and antigen retrieval was performed in citrate buffer at pH 6.0. The sections were then blocked with 10% normal goat serum (MilliporeSigma, Burlington, MA, USA; Cat# G9023) in 1% BSA, in PBS supplemented with 0.125% Triton X-100 (PBST) for 1 hour. Primary antibody in wash solution (1% BSA/1% normal goat serum in PBST) was added and sections were incubated overnight at 4°C in a humidified chamber. Sections were then washed before being incubated in secondary antibody for 1 hour at room temperature. Sections were washed with PBS and a cover slip placed using Prolong Gold Antifade with DAPI (Thermo Fisher Scientific, Waltham, MA, USA; Cat# P36935). All antibodies used for immunofluorescence are listed in Table 1. Images were obtained using a Leica DM6000 microscope equipped with a 40X objective (Leica Microsystems, Wetzlar, Germany).

### 2.3 Immunoblot

Whole mouse retinas were obtained from freshly euthanized animals using the cut-and-pick method (20) and homogenized in RIPA lysis buffer supplemented with protease and phosphatase inhibitors. Immunoblots were performed as previously described (21). Homogenized retinal tissue was centrifuged at 10,000xg for 10 min at 4°C and the supernatant was transferred to a fresh tube. The amount of protein in each sample was estimated using the BCA Protein Assay kit (Thermo Fisher; Cat# 23225). Equivalent micrograms of protein for each sample were diluted with 4X Laemmli sample buffer (Bio-Rad Laboratories, Hercules, CA; Cat# 1610747) supplemented with β-mercaptoethanol (MilliporeSigma; Cat# M6250) and heated at 95°C for 5 mins and then loaded onto a 4–20% Mini-PROTEAN^®^ TGX^™^ Precast Protein Gel (Bio-Rad; Cat# 4561094). Following electrophoresis, protein was transferred to a PVDF membrane using the TurboBlot transfer system (Bio-Rad; Cat# 1704150). Membranes were blocked with 5% non-fat milk in TBST (Tris-buffered Saline (Bio-Rad; Cat# 1706435) supplemented with Tween-20 (Thermo Fisher; Cat# 28320)) before the application of the primary antibody overnight at 4°C. Membranes were washed with TBST before adding the appropriate secondary antibody diluted in 5% milk for 1 hour at room temperature. All antibodies and their dilutions are listed in Table 1. Immunoblots were developed using SuperSignal^™^ West Dura/Femto Extended Duration Substrate (Thermo-Fisher; Cat# 34075 and 34094) and imaged using an Azure c500 imaging system (Azure Biosystems; Dublin, CA USA). Western blot bands were quantitated using ImageJ.

### 2.4 Histology and image analysis

Retinal sections through the optic nerve were selected and stained with hematoxylin and eosin for outer nuclear layer (ONL) cell counts and retinal area measurements. Retinal images for counting were acquired on a Leica DM6000 microscope with a 20X objective. The total number of nuclei in the ONL region and the total area of the ONL and the retina (from the outer edge of the ONL to the inner limiting membrane) were measured on entire sections through the plane of the optic nerve using a macro program in ImageJ as previously described (22). PR inner and outer segments were not included in the total retinal area measurement due to retraction or stretching that may occur during tissue processing, which can artificially change area measurements. The ONL cell counts were normalized to the total inner retinal area of each section to account for differences in angles of sectioning.

### 2.5 OCT and ERG

Mice were anesthetized using an intraperitoneal injection of ketamine (90 mg/kg body weight) and xylazine (10 mg/kg body weight). Their eyes were dilated using 1% tropicamide and 2.5% phenylephrine ophthalmic drops. Thickness of retinal cell layers were measured by performing optical coherence tomography (OCT) using an Envisu-R SD-OCT imager (Leica Microsystems Inc., Buffalo Grove, IL, USA) as previously described (23). Briefly, a 1.5 mm B-scan and a 1.5mm × 1.5 mm rectangular volume scan were obtained. Frames were registered and averaged using the builtin software, and the average combined inner segment and outer segment length (IS/OS), and the outer nuclear layer (ONL) measurements were determined at 16 points, spaced 140 μm apart starting at the optic nerve head according to the 9×9 template in the Diver software. For electroretinogram (ERG) measurements, mice were dark-adapted overnight and prepared as described above for ERG analysis. ERG was assessed using a Diagnosys Celeris ERG instrument (Diagnosys LLC, Lowell, MA, USA). Scotopic and photopic responses were measured as previously described (22).

### 2.6 Quantitative real-time PCR

Whole retinas were harvested as described above and immediately placed in RNAlater (Qiagen, Hilden, Germany; Cat# 76104). The RNeasy Mini Kit (Qiagen; Cat# 74104) was used to extract total RNA following the manufacturer’s protocol, and the quantity and quality of this RNA were estimated using a Nanodrop 1000 (Thermo Fisher). One microgram of total RNA was used as input for cDNA synthesis using the RNA QuantiTect Reverse transcription kit (Qiagen; Cat# 205311). 10 ng of cDNA was used as a template for each qRT-PCR reaction using the PowerTrack SYBR Green supermix (Applied Biosystems, Waltham, MA, USA; Cat# A46109). The Ct values for *Actb* were used to determine relative transcript expression levels using the 2^ΔΔCt^ method. Supplementary Table 1 contains a list of all primers and associated genes assayed. *Got1* primers used in the rod PR-specific cKO retina were located on exons 6 (forward) and 7 (reverse).

### 2.7 Targeted metabolomics

Whole mouse retinas were harvested as described above and processed for targeted metabolomics as previously described (21, 24, 25). Briefly, both retinas from each animal were washed in PBS to remove adhered vitreous, pooled, and snap-frozen before processing. The tissue was homogenized in ice-cold 80% methanol using an OMNI Bead Ruptor (OMNI International, Kennesaw, GA, USA; Cat# 19–050A). Lysates were centrifuged at 14,000g for 10 minutes at 4°C, and the supernatant was stored at −80°C until further processing. To determine the protein concentration for each sample, parallel retinas were collected and processed as described above to recover total protein. The protein concentration was determined using the Pierce^™^ BCA Protein Assay Kit (Thermo Fisher; Cat# 23225). The protein concentration for each sample was used to normalize the amount of input metabolite. The appropriate amount of supernatant was transferred to a fresh micro-centrifuge tube and lyophilized with a SpeedVac concentrator (Thermo Fisher; Cat# 13875355). These dried metabolite pellets were resuspended and subjected to liquid chromatography-coupled mass spectrometry (LC/MS) analysis using an Agilent Technologies Triple Quad 6470 instrument (Santa Clara, CA, USA).

Previously published parameters were used for data collection (24–26). Agilent MassHunter Workstation Quantitative Analysis Software (B0900) was used to process raw data. Additional statistical analyses were performed in Microsoft Excel. Each sample was normalized by the total intensity of all metabolites to reflect sample protein content. To obtain relative metabolites, the metabolite abundance level in each sample was divided by the mean of the abundance levels across all control samples.

### 2.8 Statistical analysis

Results are expressed as mean ± SEM. All sample numbers and explanations for significant values are presented in the figure legends. Groups of 2 were analyzed using a two-tailed student’s t-test. Statistical analysis was performed using GraphPad Prism version 10.0.0 for Windows.

## 3 Results

### 3.1 GOT1 is essential for photoreceptor survival

**FIGURE 1:**
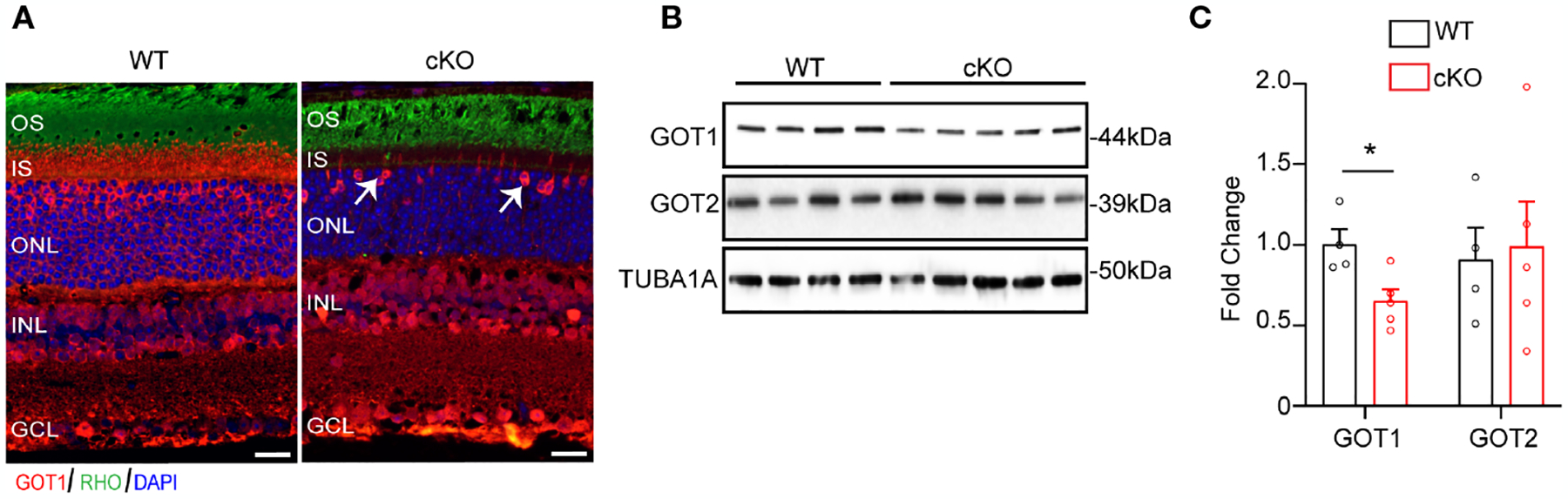
Successful deletion of GOT1 from rod photoreceptors. (A) GOT1 immunofluorescence (red) showing normal GOT1 staining in WT (*Got1*^*+*^*;Rho-Cre*^*+*^) mice. GOT1 staining is significantly reduced in the outer neuroretina and restricted to cone photoreceptors (white arrows) in the outer nuclear layer (ONL) of cKO (*Got1*^*fl/fl*^*;Rho-Cre*^*+*^) mice. Scale bar = 10 μm. OS-outer segment; IS-inner segment; INL-inner nuclear layer; GCL-ganglion cell layer. (B) Western blot showing decreased levels of GOT1 in the cKO retina with levels of GOT2 unchanged in WT and cKO retina. (C) Quantitative analysis of the Western blot depicted in (B) for GOT1 and GOT2 normalized to α-Tubulin (TUBA1A) in WT mice. N=4–5 animals per group; Unpaired two-tail student’s T-test as compared to WT mice; * - *P*<0.05. Graph shows mean ± SEM.

To evaluate if GOT1 is required for PR development, a rod PR-specific, *Got1* conditional knockout mouse model (cKO) was generated. To confirm loss of GOT1 protein from rod PRs, immunofluorescent staining on retinal sections from 2 month old cKO and WT animals was performed (Figure 1A). These data show loss of GOT1 expression in rod inner segments and cell bodies specifically, with cones maintaining GOT1 expression (Figure 1A, white arrows). In accordance with the immunofluorescence data, western blot analysis revealed a significant decrease in the expression of GOT1 protein compared to WT animals (Figure 1B). Quantification of these blots show an approximately 35% decrease in GOT1 protein expression (Figure 1C). These data also show that animals lacking GOT1 in rod PRs do not significantly upregulate the GOT2 (mitochondrial) isoform (Figures 1B, C).

**FIGURE 2:**
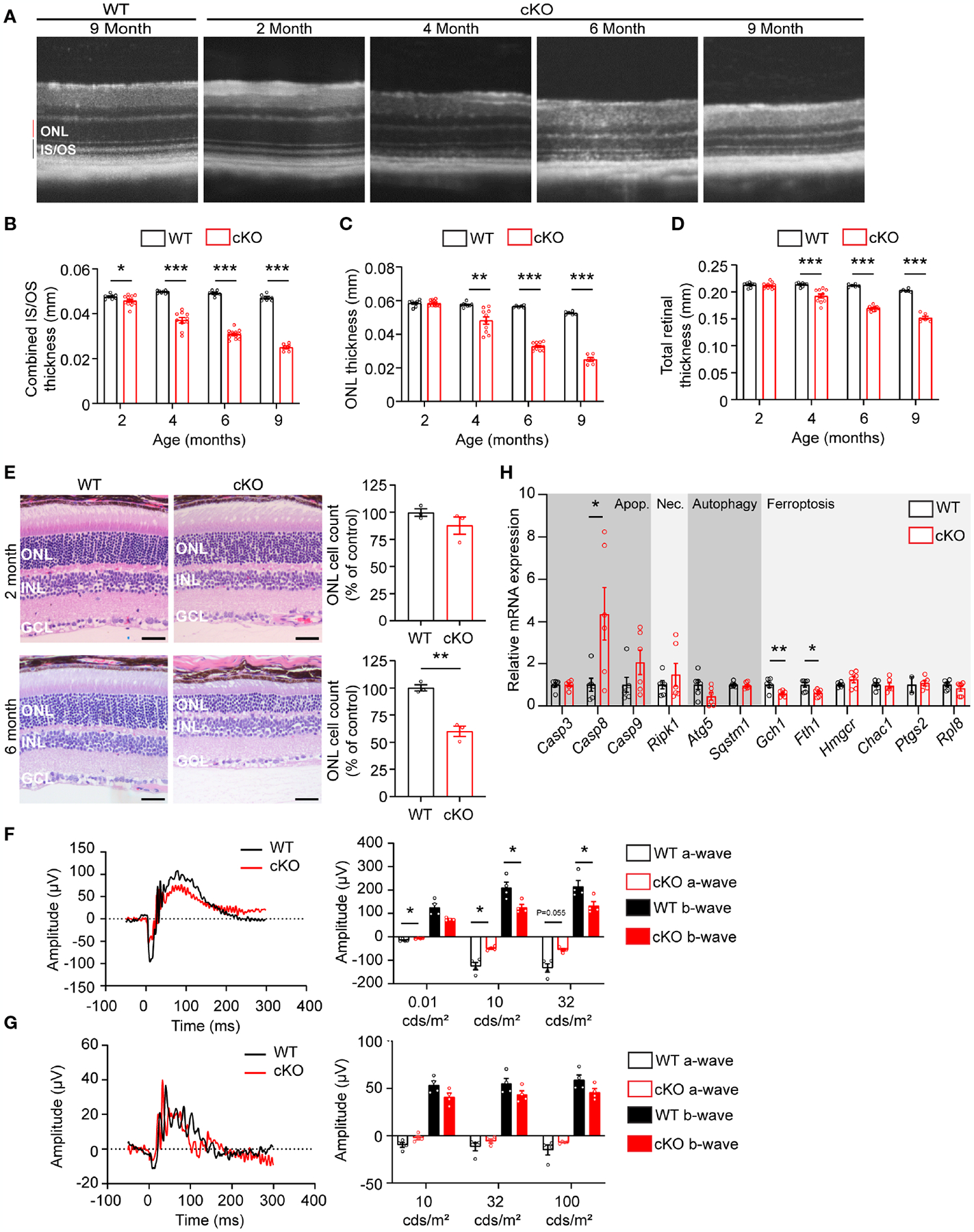
*Got1* knockdown causes progressive retinal degeneration. **(A)** Representative OCT images demonstrate progressive outer retinal degeneration in the cKO (*Got1*^*fl/fl*^*;Rho-Cre*^*+*^) mice as compared to the WT (*Got1*^*+/+*^*;Rho-Cre*^*+*^) mice. Quantitative analysis of the OCT images revealed a decrease in the **(B)** combined inner segment/outer segment (IS/OS) thickness, **(C)** outer nuclear layer (ONL) thickness and **(D)** total retinal thickness over a time course of 2 to 9 months in the cKO mice (red bars) as compared to the WT mice (black bars). N=4–5 animals per group. **(E)** Histology confirmed the lack of change in ONL cells in cKO mice (red bars) at 2 months of age as compared to the WT mice (black bars), and the significant loss of ONL cells in the cKO mice at 6 months of age. N=3 animals per group. **(F)** Representative scotopic ERG (32 cds/m^2^) traces and associated quantitation (right) showing a decrease in both the a- and b-wave amplitudes of cKO animals by 4 months of age. **(G)** Representative photopic ERG (1 Hz 100 cds/m^2^) traces and quantitation (right) showing no statistically significant change in either the a- or b-wave amplitudes in the cKO animals by 4 months of age. N=4 animals per group. **(H)** qRT-PCR of genes related to cell death pathways including apoptosis (Apop.), necroptosis (Nec.), autophagy and ferroptosis. N=6 animals per group. Unpaired two-tail student’s T-test as compared to WT mice; * - P<0.05, ** - P<0.01 and *** - P<0.001. Graphs show mean ± SEM.

To determine if GOT1 protein is required for long-term PR survival, cKO and WT mice were assessed for retinal thickness using optical coherence tomography (OCT) out to 9 months of age. These data show that loss of GOT1 leads to statistically significant thinning of the inner segment/outer segment (IS/OS) layer by 2 months of age without any decrease in outer nuclear layer thickness (Figures 2A–C). At 4 months of age, a significant thinning of the ONL becomes apparent along with progressive thinning of the IS/OS (Figure 2B). This thinning progressed as animals aged with an approximately 50% decrease in ONL thickness by 6 months (Figure 2C). Significant decreases in total retinal thickness were observed in cKO animals starting at 4 months (Figure 2D) and histological analysis confirmed the loss of PRs from the ONL at 6 months of age (Figure 2E). In addition, glial fibrillary acidic protein (GFAP) staining at 6 months of age confirmed no activation of Müller glia cells (Supplementary Figure 1).

Loss of PR cells typically leads to a loss of function as measured by ERG. To confirm that PR loss is resulting in functional changes, 4-month-old cKO and WT retinal function was assessed using ERG. Under scotopic conditions, a difference in both a- and b-wave amplitudes were observed (Figures 2F, G). Under photopic conditions, ERG analysis did not show any statistically significant changes, indicating that cone function is unchanged at this timepoint.

The decrease in PR survival and function associated with aging in the cKO animals suggested activation of cell death pathways. To assess any molecular changes related to cell death pathways, qRT-PCR was performed on retinas from 2-month-old cKO and WT mice, prior to any significant thinning of the ONL. Genes involved in apoptosis, necroptosis, ferroptosis, and autophagy were assessed (Figure 2H). *Casp8*, a marker of apoptosis, was significantly upregulated in the cKO retinas with *Casp9* demonstrating an increasing trend without reaching statistical significance. *Ripk1*, a marker of necroptosis, also showed an increasing trend in cKO animals. Two genes essential for defense against ferroptotic cell death, *Gch1* and *Fth1*, were significantly decreased potentially rendering PRs susceptible to ferroptosis as well (27, 28).

### 3.2 Loss of GOT1 alters retinal metabolism

**FIGURE 3:**
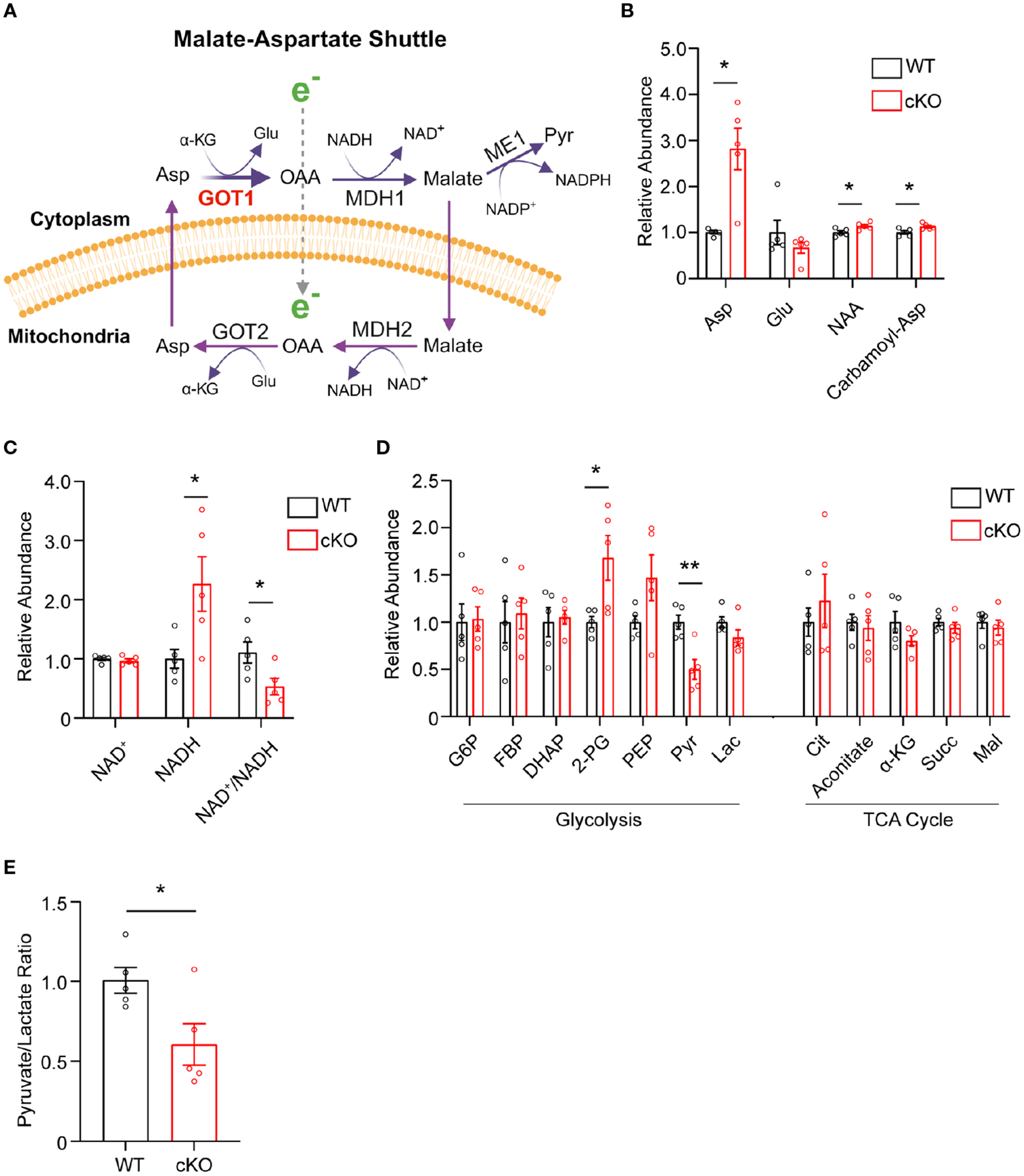
*Got1* knockdown dysregulates retinal metabolism. **(A)** Schematic depicting GOT1 within the malate-aspartate shuttle. **(B)** Relative abundance of Asp and its downstream metabolites in the retina of the WT (*Got1*^*+/+*^*;Rho-Cre*^*+*^) mouse (black bars) versus the retina of the cKO (*Got1*^*fl/fl*^*;Rho-Cre*^*+*^) mouse (red bars). **(C)** Relative abundance of NAD^+^ and NADH and the ratio NAD^+^/NADH in the retina of the WT mouse versus the retina of the cKO mouse. **(D)** Relative abundance of metabolites in glycolysis and the TCA cycle. **(E)** Pyruvate to lactate ratio of WT and cKO mouse retina at 2 months of age. N=5 animals per group; Unpaired two-tail student’s T-test as compared to WT mice; * - *P*<0.05 and ** - P<0.01. Graphs show mean ± SEM. Metabolite abbreviations include alpha-ketoglutarate (α-KG), oxaloacetate (OAA), glutamate (Glu), aspartate (Asp), N-acetylaspartate (NAA), carbamoyl-aspartate (Carbamoyl-Asp), nicotinamide adenine dinucleotide (NAD^+^), nicotinamide adenine dinucleotide + hydrogen (NADH), glucose-6 phosphate (G6P), fructose 1,6-bisphosphatase (FBP), dihydroxyacetone phosphate (DHAP), 2-phosphoglycolate (2-PG), phosphoenolpyruvate (PEP), pyruvate (Pyr) and lactate (Lac), citrate (Cit), succinate (Succ) and malate (Mal).

The data presented thus far demonstrate that GOT1 is essential for normal PR survival and function. Previous studies have shown that MAS function (Figure 3A) in the retina is critical for glucose, amino acid, and mitochondrial metabolism (8, 12, 13). However, the importance of GOT1 in maintaining the MAS and metabolic homeostasis specifically within PRs *in vivo* is not known. To interrogate the role of GOT1 in PR metabolism, unlabeled targeted metabolomics was performed on retina harvested from 2-month-old WT and cKO mice before significant PR degeneration (Supplementary Table 2). Consistent with GOT1 knockdown, a near 3-fold increase in aspartate was observed in the cKO retina without a significant change in glutamate (Figure 3B). As aspartate is a biosynthetic precursor of carbamoyl-aspartate and N-acetylaspartate (NAA), it is not surprising that a small but statistically significant increase was observed in these metabolites as well in the cKO retina (Figure 3B). Additionally, a greater than 2-fold increase in NADH was observed without any change to NAD^+^ in the retina of cKO mice as compared to WT, leading to a significant reduction in the NAD^+^/NADH ratio in the cKO retina (Figure 3C). Interestingly, only a few metabolites in glycolysis and the TCA cycle were significantly altered at 2 months of age in the cKO retina with pyruvate demonstrating a 2-fold decrease (Figure 3D). Accordingly, the pyruvate/lactate ratio was decreased in the *Got1* cKO retina (Figure 3E), which is consistent with the NAD^+^/NADH ratio.

### 3.3 GOT1 knockdown changes the expression of genes involved in metabolism and redox balance

**FIGURE 4:**
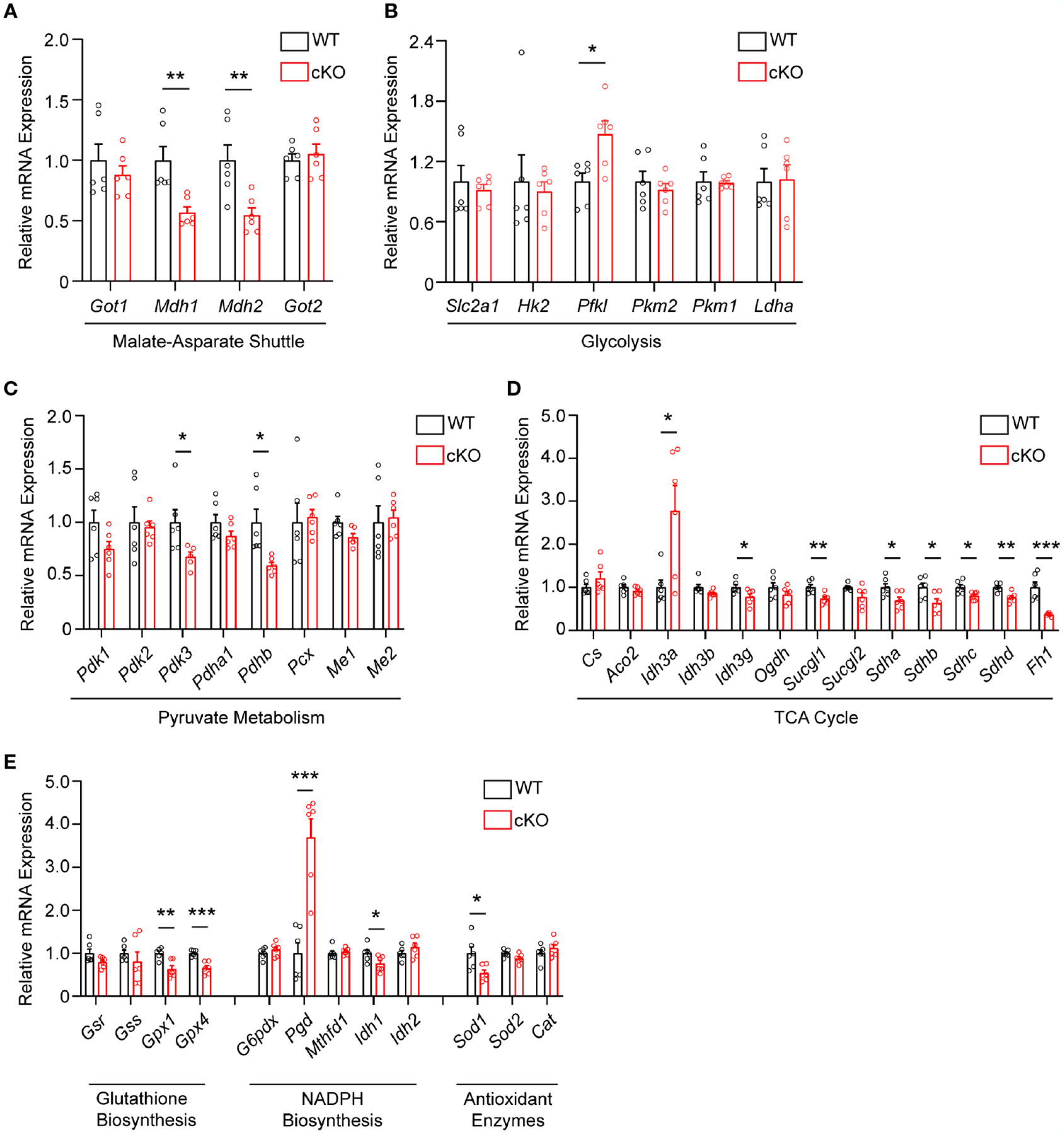
Metabolic and redox balance gene expression is altered in *Got1* cKO retina. qRT-PCR of genes related to the **(A)** Malate-Aspartate Shuttle, **(B)** Glycolysis, **(C)** Pyruvate Metabolism, **(D)** TCA cycle, and **(E)** Glutathione Biosynthesis, NADPH Biosynthesis and Antioxidant Enzymes in WT (*Got1*^*+/+*^*;Rho-Cre*^*+*^; black bars) compared to cKO (*Got1*^*fl/fl*^*;Rho-Cre*^*+*^; red bars) retina. N=6 animals per group; Unpaired two-tail student’s T-test as compared to WT mice; * - *P*<0.05, ** - *P*<0.01 and *** - *P*<0.001. Graphs show mean ± SEM.

As disruption of the MAS and resultant alteration of the NAD^+^/NADH ratio has been shown to have widespread effects on central glucose metabolism, mitochondrial function, and redox balance (9, 29, 30), the expression of genes involved in the MAS, glycolysis, pyruvate metabolism, the TCA cycle, and redox balance were examined in 2-month-old WT and cKO retina using qRT-PCR (Supplementary Table 1). With regards to the MAS, *Got1* and *Got2* expression was unaltered in the cKO retina but the expression of the NAD-dependent malate dehydrogenases (*Mdh1* and *Mdh2*) was significantly reduced in the cKO retina as compared to the WT (Figure 4A). In accordance with the few changes observed in glycolytic metabolites, few statistically significant changes were noted in the expression of genes involved in glycolysis (Figure 4B). Consistent with pyruvate being the glycolytic metabolite with the greatest change in the cKO retina (Figure 3D), genes associated with pyruvate metabolism, such as *Pdhb* and *Pdk3*, were significantly altered in the cKO retina (Figure 4C). While no significant TCA cycle metabolite changes were noted in the 2-month-old cKO retina (Figure 3D), several genes important to the TCA cycle demonstrated changes in their expression. For example, *Idh3g*, which utilizes NAD^+^, as well as *Suclg1, Sdha, Sdhb, Sdhc, Sdhd*, and *Fh1* were all found to be significantly downregulated in the cKO retina (Figure 4D).

GOT1 has been shown to be essential for redox balance and survival in pancreatic cancer cells through the generation of NADPH (9, 30). Considering the metabolic similarities between cancer cells and PRs (8, 31), we assessed changes in the expression of genes involved in key redox balance pathways such as glutathione biosynthesis, NADPH metabolism, and antioxidant enzymes in 2-month-old cKO and WT retina. The glutathione peroxidases, *Gpx4* and *Gpx1*, were significantly downregulated in the cKO retina (Figure 4E). Consistent with a possible lack of NADPH production after loss of GOT1, *Pgd*, the gene that encodes for a NADPH-producing enzyme in the oxidative pentose phosphate pathway (9), was upregulated by 3.6 fold. Additionally, *Sod1*, which encodes for the enzyme responsible for neutralizing superoxide radicals, was also significantly downregulated.

## 4 Discussion

In this study, we provide evidence that GOT1 activity is essential for rod PR function and survival *in vivo*. Rod-specific loss of GOT1 resulted in a decrease in IS/OS length followed by PR degeneration and loss of function in an age-related manner. Data presented here suggests that loss of GOT1 activity in rod PRs disrupts the MAS and alters the NAD^+^/NADH ratio to potentially affect multiple aspects of metabolism including mitochondrial function and redox balance, both of which contribute to PR degeneration (6).

Disrupting the MAS at the cytosolic GOT1 step would be expected to increase the level of cytosolic aspartate with concomitant reduction in the cytosolic NAD^+^/NADH ratio (Figure 3A). At the same time, loss of GOT1 function should increase the NAD^+^/NADH ratio within the mitochondria (29). Indeed, aspartate was significantly increased and the NAD^+^/NADH ratio was decreased overall in the GOT1 cKO retina suggesting a disruption in the MAS. Glycolysis requires NAD^+^ for continued flux; however, few changes were noted in glycolytic metabolite or gene expression levels for the cKO retina at 2 months of age, and NAD^+^ levels were unchanged at this age in the cKO retina. Considering the lactate dehydrogenase reaction is in equilibrium with the cytosolic NAD^+^/NADH ratio and the pyruvate/lactate ratio was decreased, it is likely that pyruvate reduction to lactate is maintaining cytosolic NAD^+^ to support glycolysis. The unchanged lactate level in the cKO retina may be secondary to increased export from the PRs and uptake by the retinal pigment epithelium and choroid (32). There is also an alternative cytosolic NAD^+^-regenerating pathway through glycerol 3-phosphate dehydrogenase that catalyzes the formation of glycerol 3-phosphate (G3P). A trend towards increased G3P was observed in the unlabeled targeted metabolomics for the cKO retina as compared to the WT, but it did not reach statistical significance (fold change=1.3, p=0.15; data not shown).

Loss of GOT1 in rod PRs disrupts the MAS and likely hinders the ability of PRs to transfer reducing equivalents across the mitochondrial membrane, resulting in an increased NAD^+^/NADH ratio within the mitochondria. Previous studies have demonstrated that disrupting the MAS impairs mitochondrial fitness as indicated by a reduction in mitochondrial oxygen consumption (29). While oxygen consumption was not assayed here, qRT-PCR analysis did reveal a multitude of changes in genes encoding for several enzymes involved in the TCA cycle and electron transport chain, suggesting that mitochondrial function may be altered. *Idh3a* is one of these TCA cycle genes that demonstrated a significant change in its expression. The NAD-specific, isocitrate dehydrogenase (IDH3) has been shown to be important for photoreceptor survival as mutations in IDH3 result in retinal degeneration in humans as well as mice with mutant *Idh3a* cells demonstrating mitochondrial dysfunction (33, 34). Considering the IDH3 reaction produces mitochondrial NADH, which is expected to be reduced in the *Got1* cKO, its upregulation may signal an important compensatory response in this mouse model. Interestingly, though, the other TCA cycle enzymes involved in mitochondrial NADH production, such as *Ogdh* and *Mdh2*, were not upregulated in a similar manner.

**FIGURE 5:**
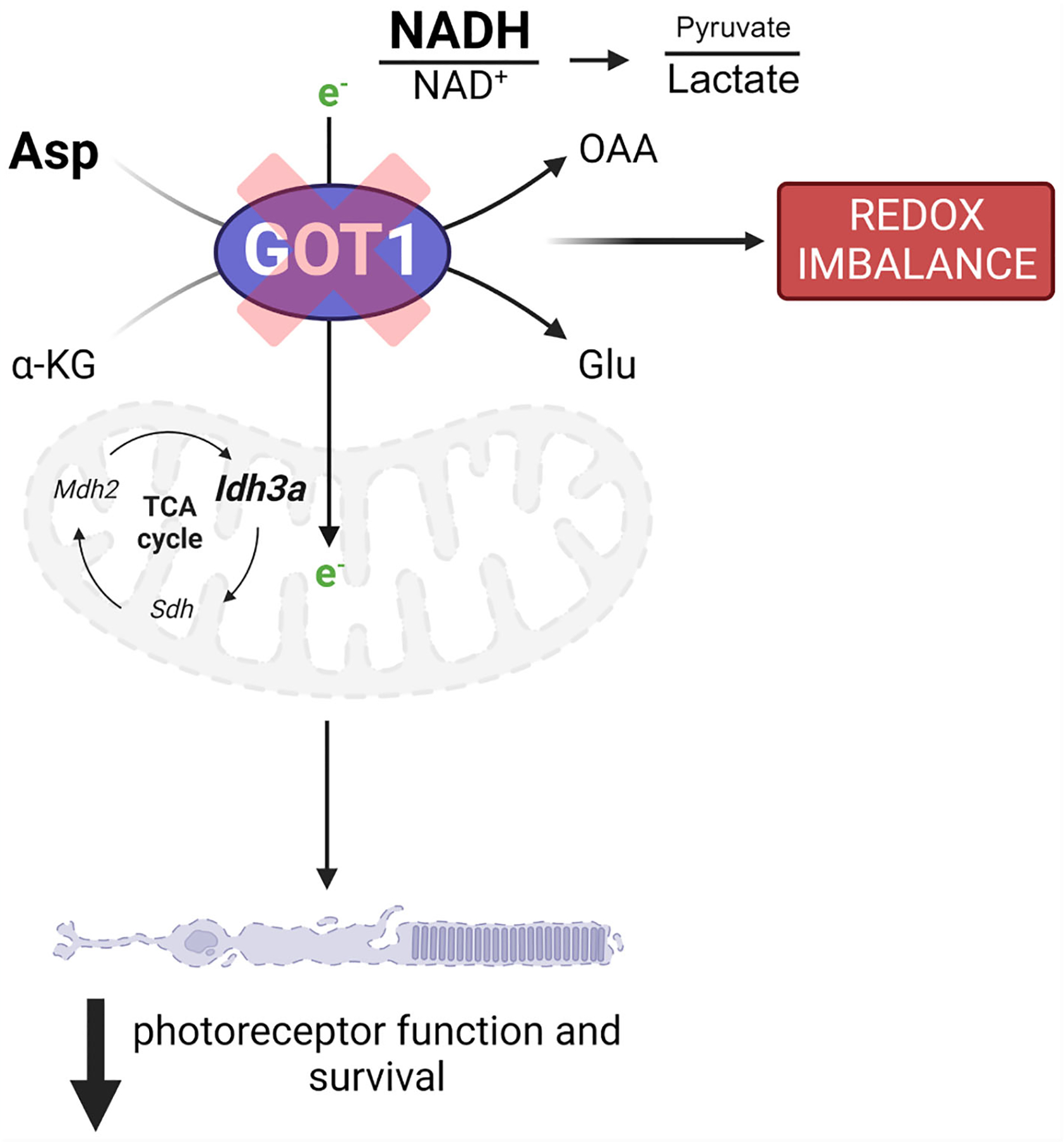
GOT1 is critical to *in vivo* photoreceptor metabolism, function, and survival. *Got1* knockdown alters the malate-aspartate shuttle resulting in increased levels of aspartate as well as the NADH/NAD^+^ ratio and a decreased pyruvate/lactate ratio. This disruption in metabolic homeostasis is characterized by alterations to TCA cycle and redox homeostasis gene expression. Ultimately, these molecular changes produce an age-related decline in photoreceptor function and survival.

Loss of GOT1 in rod PRs also resulted in decreased pyruvate levels in the retina. As discussed above, this decrease in pyruvate may be secondary to its increased conversion to lactate to maintain cytosolic NAD^+^ and glycolysis. Although only a small percentage of glucose-derived pyruvate enters the mitochondria to be oxidized to acetyl-CoA in the retina, prior studies have demonstrated that this pyruvate transport is essential for mitochondrial fitness and PR function and survival (35). As such, if less pyruvate is available to be oxidized in the mitochondria because it is instead being reduced to lactate, mitochondrial metabolism may become dysfunctional. Alterations to mitochondrial function have been reported to result in retinal degeneration and have been associated with PR cell loss in the *rd1* mouse model (35–37). While future studies are needed to assess mitochondrial oxygen consumption and the utilization of glucose carbons via stable isotope tracing metabolomics in this novel transgenic mouse model, our data suggest that GOT1 knockdown and its effects on the NAD+/NADH ratio and pyruvate may result in perturbed mitochondrial function, which in turn can lead to PR degeneration (Figure 5).

Maintaining redox homeostasis is crucial for PR survival and function (6). The expression of genes encoding enzymes responsible for maintaining redox balance were altered in the cKO as compared to the WT retina. Of these, the expression of *Pgd* was increased nearly 4-fold. *Pgd* encodes for 6-phosphogluconate dehydrogenase (PGD), which produces NADPH in the oxidative pentose phosphate pathway. PGD function has been shown to be critical for metabolic and redox homeostasis in cells deficient in oxidative phosphorylation (38). GOT1 has been shown to be critical for maintaining mitochondrial oxidative phosphorylation as well as redox balance in pancreatic cancer cells through the production of NADPH, and ME1 is required for the generation of NADPH in this pathway (9, 26, 30). Interestingly, ME1 has been implicated in controlling glutathione content in the retina (39). It is possible that knockout of GOT1 in rod PRs impairs mitochondrial oxidative phosphorylation considering the decreased expression of genes that make up complex II (*Sdha, Sdhb, Sdhc, Sdhd*) and alters NADPH biosynthetic pathway through ME1, for which PRs may try to compensate by increasing the expression of *Pgd*. Future studies are necessary to investigate if ME1 function is required for PR homeostasis, or if other pathways can be enhanced to compensate for the metabolic and redox disturbances that result from GOT1 knockdown.

Previous *in vivo* work investigating the importance of the MAS in the retina focused on the mitochondrial aspartate-glutamate transporter Aralar (AGC1); however, *Agc1* knockout only resulted in a functional change without loss of PR cells (12). This report investigated a full retinal knockout and only looked at retinal structure at postnatal day 17. The data presented in our study indicate an age-related PR cell loss when the MAS is disrupted, so it cannot be ruled out that degeneration may occur at a later timepoint in the *Agc1* knockout animal. Investigation into the metabolism of *ex vivo* retina from *Agc1* knockout animals showed decreased pyruvate, an increased lactate/pyruvate ratio, and a decrease in aspartate and glutamine levels (8, 12, 40, 41). These data are consistent with the metabolomics data herein as a lack of aspartate transfer from the mitochondria to cytoplasm would decrease the cytosolic NAD^+^/NADH ratio. To regenerate NAD^+^, cells would need to reduce pyruvate to lactate, increasing the lactate/pyruvate ratio. In contrast to the *Agc1* knockout, our *in vivo* metabolomics data demonstrated an increase in aspartate and a small increase in NAA when *Got1* is deleted from PRs, whereas *Agc1* knockout shows decreased aspartate and decreased NAA as well as glutamine. The loss of AGC1 should trap aspartate in the mitochondria, whereas loss of GOT1 should result in aspartate accumulation in the cytoplasm. PRs have a symbiotic relationship with Müller glia (MG) where aspartate from PRs is shuttled to MG, which subsequently generate glutamine for PR utilization (41). Therefore, it is not surprising that we did not see any decrease in glutamine as based on this proposed symbiotic relationship, MG will have ample amounts of aspartate at their disposal. Additionally, *ex vivo* studies have demonstrated that aspartate aminotransferase and an intact MAS are important for the production and maintenance of the glutamate pool in the retina (8, 13, 14). Yet, no significant change in glutamate was detected in GOT1 cKO mouse retina as compared to the WT suggesting potential metabolic rewiring to compensate for decreased aspartate aminotransferase activity. Another major pathway for glutamate synthesis is through glutaminase, which converts glutamine to glutamate. Future work using stable isotope tracing metabolomics will help unravel which pathways are being used to compensate for the loss of GOT1 in cKO animals.

In conclusion, the data presented here unveil a necessary role for the MAS and GOT1 in maintaining PR health and function *in vivo*. It will be important to assess the different roles of the cytosolic aspartate aminotransferase, GOT1, and the mitochondrial aspartate aminotransferase, GOT2, and how they relate to the MAS to promote PR function and survival. The insights gained on the biological role of this pathway and potential compensatory pathways in PRs will provide a foundation for developing novel metabolism-based therapies for retinal degenerative disease.

## Supplementary Material

Supp Fig1

Supp Table1

Supp Table2

## Figures and Tables

**TABLE 1:** Antibodies used in this study.

Protein target	Dilution	Supplier (Catalog number)
**GOT1**	1:200 (IF), 1:2000(WB)	Abcam (ab239487)
**GOT2**	1:2000 (WB)	Atlas antibodies (HPA 018139)
**GFAP**	1:200 (IF)	Thermo Fisher (13-300)
**Rhodopsin (RHO)**	1:1000 (IF)	Abcam (ab5417)
**TUBA1A**	1:5000 (WB)	MilliporeSigma (T6199)
**Alexa 594 Anti-mouse**	1:500 (IF)	Jackson ImmunoResearch Laboratories (715-585-151)
**Alexa 488 Anti-mouse**	1:1000 (IF)	Invitrogen (A11001)
**Anti-mouse IgG, HRP-linked**	1:5000 (WB)	Cell Signaling Technology (7076)
**Anti-Rabbit IgG, HRP-linked**	1:5000 (WB)	Cell Signaling Technology (7074)

IF, Immunofluorescence; WB, Western Blot; HRP, Horse Radish Peroxidase.

## Data Availability

The original contributions presented in the study are included in the article/Supplementary Materials, further inquiries can be directed to the corresponding author.
