# Supplementary figures and images for: Rod photoreceptor-specific deletion of cytosolic aspartate aminotransferase, GOT1, causes retinal degeneration
Source: Front Ophthalmol (Lausanne). Author manuscript; Available in PMC 2024 May 9. (PMC11081273; doi:10.3389/fopht.2023.1306019)

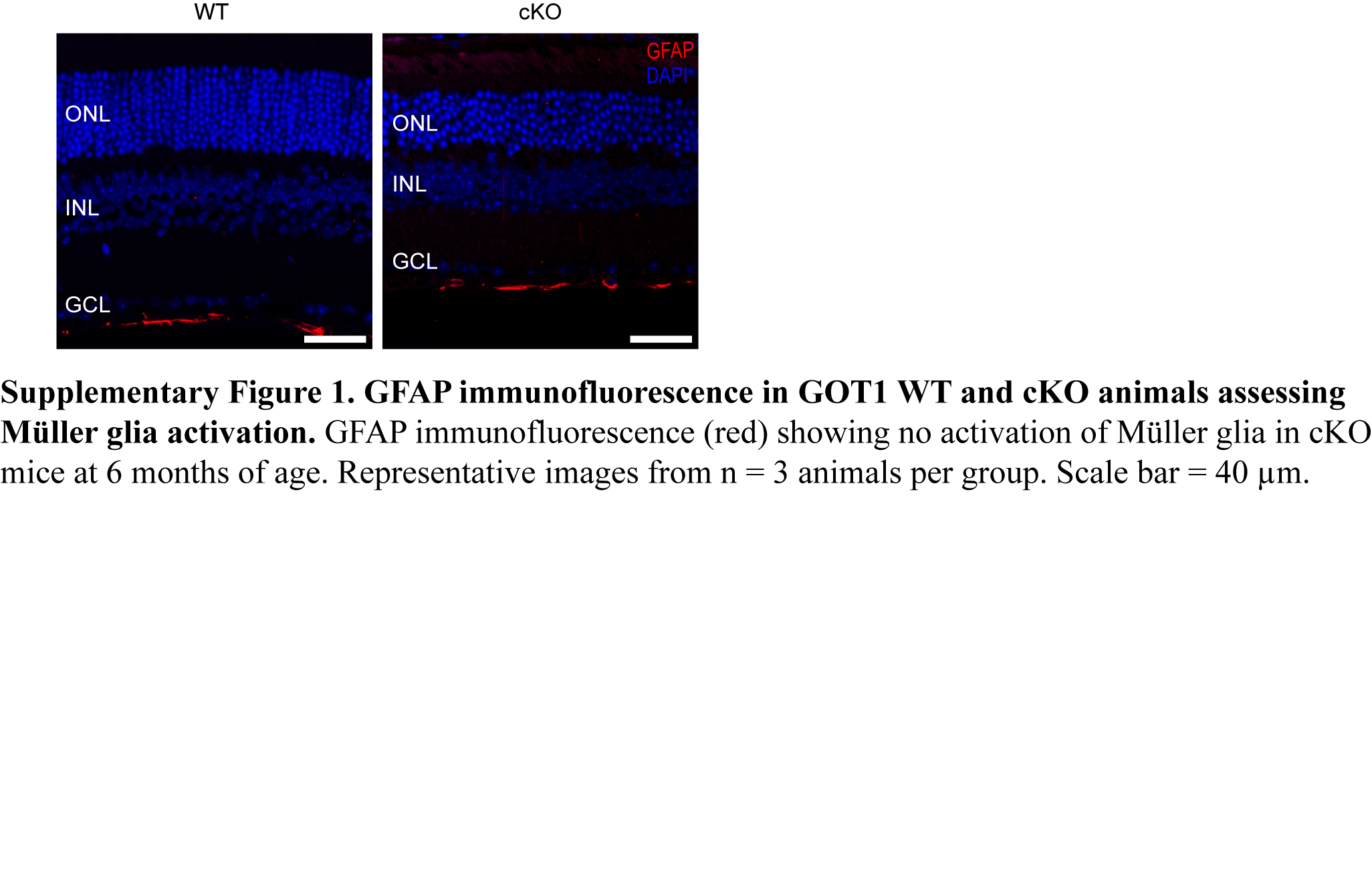

Supplement: Supp Fig1 [file NIHMS1984497-supplement-Supp_Fig1.tif]
