# Supplementary material for: Rod photoreceptor-specific deletion of cytosolic aspartate aminotransferase, GOT1, causes retinal degeneration
Source: Front Ophthalmol (Lausanne). Author manuscript; Available in PMC 2024 May 9. (PMC11081273; doi:10.3389/fopht.2023.1306019)
Supplement: Supp Table1 [file NIHMS1984497-supplement-Supp_Table1.docx]

| **Supplementary Table 1. Gene expressions changes in cKO versus WT retina at 2 months of age.** | | | | | |
| --- | --- | --- | --- | --- | --- |
| **Pathway** | **Gene Abbreviation** | **Forward Primer** | **Reverse Primer** | **Fold Change** | **P-value** |
| Reference Gene | *Actb* | AGCCATGTACGTAGCCATCC | CTCTCAGCTGTGGTGGTGAA | N/A | N/A |
| Malate-Aspartate Shuttle | *Got1* | AGAGAAAGATGCGTGGGCTA | TGGACCAGGTGATTCGTACA | 0.88 | 0.461 |
|  | *Mdh1* | GAAGCCCTGAAAGACGACAG | TCGACACGAACTCTCCCTCT | 0.57 | 0.011 |
|  | *Mdh2* | GCTTTGTCTTCTCCCTCGTG | CAAAGTCCTCGCCTTTCTTG | 0.55 | 0.014 |
|  | *Got2* | GTTGAAATGGGACCTCCAGA | GGGCAGGTATTCTTTGTCCA | 1.05 | 0.620 |
| Glycolysis | *Slc2a1* (1) | TCAACACGGCCTTCACTG | CACGATGCTCAGATAGGACATC | 0.91 | 0.633 |
|  | *Hk2* | GGGACGACGGTACACTCAAT | GCCAGTGGTAAGGAGCTCTG | 0.90 | 0.736 |
|  | *Pfkl* | AACATTTACAAGCTCCTCGCCC | CAGCCTACTTCTTGCACCTGAC | 1.47 | 0.016 |
|  | *Pkm2* (1) | ATTACCAGCGACCCCACAGAA | ACGGCATCCTTACACAGCACA | 0.92 | 0.508 |
|  | *Pkm1* | ATAGCTCGGGAGGCTGAGGCA | GACTCCGTGAGAACTATCAAA | 0.99 | 0.894 |
|  | *Ldha* (2) | GTTGTTGGGGTTGGTGCTGT | TCATCTCGCCCTTGAGTTTG | 1.02 | 0.906 |
| Tricarboxylic Acid (TCA) Cycle | *Cs* | TGCCTAAGGATCCCATGTTC | TTCATCTCCGTCATGCCATA | 1.21 | 0.245 |
|  | *Aco2* | CAACATGGGTGCAGAAATTG | GTGAGCCAAGTCAGGGGTAA | 0.91 | 0.242 |
|  | *Idh3a* | GAGGTTTTGCTGGTGGTGTT | TCCTCCTGGTCCTTGAATTG | 2.78 | 0.029 |
|  | *Idh3b* | ATCTGAGCGAGGTGCAGAAT | TACGTTGGCAAACAAATCCA | 0.85 | 0.084 |
|  | *Idh3g* | TGTAAGCTCCAACGCTGATG | CACTCCTGGCAGGCTCTTAC | 0.78 | 0.038 |
|  | *Ogdh* | ACATGGCACAGTGCATCATT | ACATCTGCAGAAACCGCTCT | 0.83 | 0.215 |
|  | *Suclg1* | AGATTCCCTTGGTTGTGTGC | GGGTTGTTTGGTGAACTGCT | 0.73 | 0.006 |
|  | *Suclg2* | CTTTGGTGGGATCGTCAACT | AACAGCTTTCTTGGCTGCAT | 0.77 | 0.070 |
|  | *Sdha* | ACACAGACCTGGTGGAGACC | GGATGGGCTTGGAGTAATCA | 0.69 | 0.026 |
|  | *Sdhb* | ACTGGTGGAACGGAGACAAG | TTAAGCCAATGCTCGCTTCT | 0.64 | 0.025 |
|  | *Sdhc* | GGAGGGGTCTCTCTTTTTGG | AAGTGTCGGATCCCATTCAG | 0.80 | 0.022 |
|  | *Sdhd* | GATCCCTGCTGGGTACTTGA | AAGTAGCAAAGCCCAGCAAA | 0.76 | 0.003 |
|  | *Fh1* | AGCAATGCATATTGCTGCTG | CGCATACTGGACTTGCTGAA | 0.37 | 0.004 |
| Pyruvate Metabolism | *Pdk1* | GGCGGCTTTGTGATTTGTAT | ACCTGAATCGGGGGATAAAC | 0.75 | 0.096 |
|  | *Pdk2* | AGGAAGTCAATGCCACCAAC | GAGGGCCACCATAATCTTGA | 0.95 | 0.785 |
|  | *Pdk3* | GTGGAGTCCCACTTCGAAAA | AAAACTGGCAGCCTCTCAA | 0.68 | 0.041 |
|  | *Pdha1* | GGGACGTCTGTTGAGAGAGC | TGTGTCCATGGTAGCGGTAA | 0.87 | 0.177 |
|  | *Pdhb* | TCGAAGCCATAGAAGCCAGT | AGGCATAGGGACATCAGCAC | 0.59 | 0.021 |
|  | *Pcx* | ATGTTGTGGACGTGGCAGTA | AATCGAAGGCTGCGTACAGT | 1.05 | 0.807 |
|  | *Me1* | GGGATTGCTCACTTGGTTGT | GTTCATGGGCAAACACCTCT | 0.86 | 0.069 |
|  | *Me2* | TTGTGTTCCCTGCATGGTTA | ATACAGACGGGCAGACCAAC | 1.05 | 0.798 |

| Redox Balance | *Gsr* | CACGACCATGATTCCAGATG | CAGCATAGACGCCTTTGACA | 0.79 | 0.097 |
| --- | --- | --- | --- | --- | --- |
|  | *Gss* | GCCTCCTACATCCTCATGGA | CCACATGCTTGTTCATCACC | 0.81 | 0.445 |
|  | *Gpx1* | ATCAGTTCGGACACCAGGAG | CATTCCGCAGGAAGGTAAAG | 0.63 | 0.005 |
|  | *Gpx4* | CCGGCTACAACGTCAAGTTT | ACGCAGCCGTTCTTATCAAT | 0.66 | 0.001 |
|  | *G6pdx* | CCTACCATCTGGTGGCTGTT | CATTCATGTGGCTGTTGAGG | 1.08 | 0.337 |
|  | *Pgd* | GGGCACTTTGTGAAGATGGT | AACAGCTCTTTGCCGTCAGT | 3.69 | 0.001 |
|  | *Mthfd1* | AGCACAGTAGAGAGCGCACA | CAGGCGATCTAATGCTGACA | 1.04 | 0.579 |
|  | *Idh1* | AGGTTCTGTGGTGGAGATGC | GACGCCCACGTTGTATTTCT | 0.76 | 0.048 |
|  | *Idh2* | CCGTCTTCAGAGAGCCAATC | GAAATGGACTCGTCGGTGTT | 1.14 | 0.237 |
|  | *Sod1* | CCAGTGCAGGACCTCATTTT | CACCTTTGCCCAAGTCATCT | 0.54 | 0.025 |
|  | *Sod2* | CCGAGGAGAAGTACCACGAG | GCTTGATAGCCTCCAGCAAC | 0.88 | 0.058 |
|  | *Cat* | ACATGGTCTGGGACTTCTGG | CAAGTTTTTGATGCCCTGGT | 1.12 | 0.318 |
| Cell Death Pathways | *Casp3* | GGGCCTGTTGAACTGAAAAA | CCGTCCTTTGAATTTCTCCA | 1.00 | 0.984 |
|  | *Casp8* | CCTAGACTGCAACCGAGAGG | GCAGGCTCAAGTCATCTTCC | 4.36 | 0.043 |
|  | *Casp9* | GATGCTGTCCCCTATCAGGA | GGGACTGCAGGTCTTCAGAG | 2.05 | 0.155 |
|  | *Ripk1* | CCTGCTGGAGAAGACAGACC | CATCATCTTCCCCTCTTCCA | 1.48 | 0.433 |
|  | *Atg5* | AGATGGACAGCTGCACACAC | GCTGGGGGACAATGCTAATA | 0.45 | 0.085 |
|  | *Sqstm1* | GCTCAGGAGGAGACGATGAC | AGAAACCCATGGACAGCATC | 0.96 | 0.621 |
|  | *Gch1* | CACCAAGGGATACCAGGAGA | AGCCAATATGGACCCTTCCT | 0.58 | 0.014 |
|  | *Fth1* | CGAGATGATGTGGCTCTGAA | GTGCACACTCCATTGCATTC | 0.62 | 0.021 |
|  | *Hmgcr* | TGGAGATCATGTGCTGCTTC | GCGACTATGAGCGTGAACAA | 1.25 | 0.173 |
|  | *Chac1* | GTACGGCTCCCTAGTGTGGA | GTCTTCAAGGAGGGTCACCA | 0.97 | 0.833 |
|  | *Ptgs2* | AGAAGGAAATGGCTGCAGAA | GCTCGGCTTCCAGTATTGAG | 1.08 | 0.873 |
|  | *Rpl8* | AAGCGGACAGAGCTGTTCAT | CTGGGTTGTGGGAGATGACT | 0.83 | 0.299 |

**Supplementary Table References**

1. Rajala A, Wang Y, Soni K, Rajala RVS. Pyruvate Kinase M2 Isoform Deletion in Cone Photoreceptors Results in Age-Related Cone Degeneration. *Cell Death Dis* (2018) 9(7):737. Epub 2018/07/05. doi: 10.1038/s41419-018-0712-9.

2. Casson RJ, Wood JP, Han G, Kittipassorn T, Peet DJ, Chidlow G. M-Type Pyruvate Kinase Isoforms and Lactate Dehydrogenase a in the Mammalian Retina: Metabolic Implications. *Invest Ophthalmol Vis Sci* (2016) 57(1):66-80. Epub 2016/01/19. doi: 10.1167/iovs.15-17962.
